# Associations of Polygenetic Variants at the 11q23 Locus and Their Interactions with Macronutrient Intake for the Risk of 3GO, a Combination of Hypertension, Hyperglycemia, and Dyslipidemia

**DOI:** 10.3390/jpm11030207

**Published:** 2021-03-15

**Authors:** Jun-Yu Zhou, Sunmin Park

**Affiliations:** 1 Department of Bio-Convergence System, Hoseo University, Asan 31499, Korea; zjy888zjy888@gmail.com; 2 Department of Food and Nutrition, College of Life and Health Sciences, Hoseo University, Asan 31499, Korea

**Keywords:** hypertension, hyperglycemia, dyslipidemia, haplotype, 3GO, undigested carbohydrates, dietary fiber

## Abstract

3GO is a condition in which hypertension, hyperglycemia, and dyslipidemia co-occur, and these conditions are related to each other and genetic and environmental factors. We hypothesized that common genetic variants and their interactions with lifestyles influenced 3GO risk. We aimed to explore common genetic variants to affect 3GO risk and their haplotype interaction with lifestyles in a city hospital-based cohort in 58,701 Koreans > 40 years. 3GO was defined as SBP ≥ 140 mmHg and DBP ≥ 90 mmHg for hypertension, fasting blood glucose ≥ 126 mg/dL for hyperglycemia, and LDL ≥ 160 mg/dL or HDL ≤ 40 mg/dL, or triglyceride ≥ 200 mg/dL for dyslipidemia. Haplotypes were generated by genetic variants selected from genome-wide association study ((GWAS) an observational study of the genetic variation of the whole genome in different individuals, used to see if any variation is related to traits) after adjusting for age, sex, area of residence, and body mass index (BMI). Nutrient intakes were assessed using food frequency questionnaires. Interactions between haplotype and lifestyles and 3GO risk were investigated. Parameters related to metabolic syndrome were significantly different in the 0GO, 1–2GO, and 3GO groups, that is, groups of individuals with none, one to two, or all three of the components of 3GO. At the 11q23 locus, *KCNQ1*_rs2237892, *ZPR1*_rs2075291, *APOA5*_rs662799, *APOA1*_rs5072, and *SIK3*_rs151139277, influenced 3GO risk, and the minor alleles of their haplotype had a 3GO risk 3.23 times higher than the major alleles. For subjects with a high energy intake, the 3GO risk of the minor alleles was significantly higher than that of the major alleles (OR = 3.230, 95% confidence interval (CI) = 2.062~5.061, *p* < 0.001). BMI, HbA1c, SBP, and serum concentrations of glucose, HDL, and triglyceride were significantly higher for the minor allele than the major alleles (*p* < 0.001). The haplotype interacted with the intakes of protein (*p* = 0.033), digestible carbohydrate (*p* = 0.012), fat (*p* = 0.008), and undigestible carbohydrates (*p* = 0.015) to increase 3GO risk. An interaction was also observed between smoking and the haplotype (*p* = 0.007). The minor allele effects on 3GO incidence were higher in the high digestible carbohydrate intake and smoking groups. By contrast, the minor allele impacts on 3GO frequencies were much higher in the low intake of undigestible carbohydrates, protein, and fat. In conclusion, people who carry a minor allele of the 11q23 locus haplotype should avoid smoking and replace digestible carbohydrate intake with consuming high-quality protein, healthy fat, and undigestible carbohydrates.

## 1. Introduction 

The World Health Organization has stated that reducing metabolic syndrome (MetS) development should be considered the first defense against cardiovascular diseases [1]. The mortality rate related to cardiovascular diseases is as high as 35.7% in Asians [2]. Early-stage MetSe has the characteristics of abdominal obesity, hypertension, hyperglycemia, and dyslipidemia, and progresses to metabolic diseases such as type 2 diabetes (T2DM) and cardiovascular diseases [1]. The components of MetS also influence the development and progression of metabolic diseases. Although abdominal obesity plays a vital contributor role, Asians that develop hyperglycemia lose weight, and thus, in Asians, abdominal obesity and obesity do not significantly affect metabolic diseases [3]. Relatively lean Asians develop hyperglycemia, hypertension, and dyslipidemia [3]. 

The other three components of MetS (hypertension, hyperglycemia, and dyslipidemia) are closely interrelated and share risk factors, such as aging, menopause, obesity, inflammation, and oxidative stress [1]. Hypertension, hyperglycemia, and dyslipidemia share insulin resistance as a common mechanism [4]. According to a recent report, about 40% of diabetic patients have lipid metabolism disorders, as evidenced by high triglyceride and low high-density lipoprotein levels. T2DM causes dyslipidemia because lipoprotein lipase activity is reduced due to reduced insulin activity [5]. Moreover, those with T2DM exhibit increased lipid synthesis in the liver, which contributes to dyslipidemia and the development of non-alcoholic fatty liver [6]. Dyslipidemia increases blood vessel thickness by cholesterol deposition and thus, elevates blood pressure. Hypertension is also common in patients with T2DM and dyslipidemia [5]. Hypertension, hyperglycemia, and dyslipidemia are also substantial risk factors of cardiovascular and cerebrovascular diseases [7], and genetic and environmental factors influence the development of all three MetS components. Specific genetic variants have been reported to enhance susceptibilities to hypertension, hyperglycemia, and dyslipidemia [8,9], and some of these genetic variants are possibly shared, which suggests hypertension, hyperglycemia, and hyperglycemia should be simultaneously addressed [10]. 

Relatively non-obese Asians with MetS are susceptible to progress to T2DM, dyslipidemia, and hypertension. 3GO is a newly coined word for a syndrome comprised of hypertension, hyperglycemia, and dyslipidemia, and it is well-recognized in China since these diseases often simultaneously occur in one person [11,12]. GO corresponds to ‘high’ in the East Asian cultural sphere, including Chinese, Korean, Japanese, Vietnam, and Singapore [13], and 3 represents its three components. The cutoff values used to define 3GO are better-specified disease status than those for MetS, and 3GO indicates the co-existence of T2DM, hypertension, and dyslipidemia [14]. Accordingly, the prevention of 3GO may reduce mortality and morbidity and reduce medical costs. In this study, we hypothesized that common genetic variants influence 3GO risk and that they interact with lifestyles. Therefore, the study aimed to explore common genetic variants of 3GO risk, and the PRS of the common genetic variants interacted with lifestyles to influence 3GO risk in a city hospital-based cohort in Korean adults ≥ 40 years. The results of this study could be applied to prevent 3GO in those at elevated 3GO risk by modifying lifestyles, including smoking, drinking alcohol, nutrient intake, and exercise, according to genetic status.

## 2. Materials and Methods

### 2.1. Study Population

The epidemiological data used to represent the Korean urban population was obtained from the Korean Genomics and Epidemiology Study (KoGES) conducted by the Korean Center for Disease Control and Prevention. The Institutional Review Board of the Korean National Institute of Health (KBP-2015-055) approved the study protocol. All participants volunteered to take part in the study and provided written informed consent. Before conducting the research, we also obtained approval from the Institutional Review Board of Hoseo University (1041231-150811-HR-034-01). KoGES was initiated in 2004 and terminated in 2013 and was a population-based, prospective cohort study that recruited 58,701 Korean adults aged 40–77 years (20,293 males and 38,408 females). Follow-up surveys are conducted every two years, and study participants were followed up to 5 times during the 10-year study period. Information about lifestyles, current medications, and sociodemographic characteristics was obtained by a questionnaire-based survey.

### 2.2. Sociodemographic Characteristics and Biochemical Measurements

Questionnaires provided details of residence area, gender, age, smoking, drinking, and exercise status. Physical activity was estimated by multiplying rated physical activity intensities by times per week. Physical activity was classified as light, moderate, and heavy, based on moderate physical activity for <30, 30–120, and >120 min/week. Participants were classified as current, past, and never smokers. Never smokers had a lifetime smoking history of <100 cigarettes, and past-smokers had not smoked during the previous six months. Alcohol intake was assessed by multiplying the amount of drinking per occasion by the number of occasions per week, and daily alcohol intakes were classified as none (0 g), light (<2 g), moderate (2–20 g), or heavy (>20 g).

After at least 10 min rest upon arrival to the hospital between 7:00 a.m. and 9:00 a.m., blood pressure was measured at the upper right arm in a sitting position three times using a mercury sphygmomanometer (Baumanometer; WA Baum, Copiague, NY, USA) using a standard protocol [14,15]. There was a 5 min resting period between each measurement, and the average of three measurements was used for systolic blood pressure (SBP) and diastolic blood pressure (DBP). Blood samples were collected in a serum separator tube and two ethylenediaminetetraacetic acids (EDTA) tubes during mornings after an overnight fast for at least eight hours (drinking water was allowed) and no taking any medication in the morning before taking blood [14]. Serum and plasma were separated, aliquoted, and frozen for further assays [14]. The serum concentrations of glucose, high-density lipoprotein (HDL), triglycerides, and HbA1c were measured using a Hitachi automatic analyzer 7600. Body mass indices (BMI) were calculated by dividing body weight (kg) by height (m^2^), and obesity was defined as a BMI ≥ 25 kg/m^2^.

### 2.3. 3GO Definition

According to the International Hypertension Federation [16], the diagnostic criteria for hypertension (regardless of gender) are SBP of ≥140 mmHg and DBP of ≥90 mmHg. The diagnostic criterion for hyperglycemia was applied to T2DM criteria: a fasting serum glucose of ≥126 mg/dL and taking anti-diabetic medication daily [17,18]. Dyslipidemia was defined as serum levels of triglyceride, total cholesterol, and HDL of ≥200, ≥250, or <40 mg/dL, regardless of gender, according to the guidelines issued by the International Society of Endocrinology [19]. The participants who took medications for hyperglycemia, hypertension, and/or dyslipidemia were considered the case of each group. 3GO (case) was defined as the presence of all three components, including hypertension, hyperglycemia, and dyslipidemia, while 1–2GO indicated having one or two among 3GO components. The participants without any 3GO components were considered as 0GO (control).

### 2.4. Dietary Assessment and Dietary Patterns

Usual food intakes were assessed using a semi-quantitative food frequency questionnaire (SQFFQ) composed of 103 food items, and frequencies and average serving sizes were obtained for each food item. Serving sizes were assessed as 0.5, 1, or 2 times a reference serving size. Average intakes were calculated for all food items by multiplying frequencies and average daily serving sizes. The validity and reproducibility of the SQFFQ were confirmed using a 3-day food record as previously described [20]. Dietary patterns were generated by principal component analysis using SQFFQ results. Daily energy and nutrient intakes were calculated using Can-Pro 2.0 nutrient intake assessment software devised by the Korean Nutrition Society [21].

### 2.5. Genotyping and Quality Control

According to standard protocols, genomic DNA was extracted from peripheral blood monocytes of all participants using the QuickGene DNA whole-blood kit with QuickGene-610 L equipment (Fujifilm, Tokyo, Japan). Single nucleotide polymorphisms (SNP) were identified using a Korea Biobank array (K-CHIP), which contained 830,000 SNPs customized for Koreans (Affymetrix, Santa Clara, CA, USA) and designed by the Center for Genome Science of the Korea National Institute of Health (http://www.cdc.go.kr/CDC/eng/mobile/CdcKrContentView.jsp?cid=74266&menuIds=HOME001-NU1130-MNU1890 (accessed on 18 March 2020).). After genotyping, genotyping accuracy was assessed by Bayesian Robust Linear Modeling with the Mahalanobis Distance genotyping algorithm [22,23]. The experimental results of the Korea Biobank Array were filtered by the quality control procedures of the following exclusion criteria: low genotyping accuracy (<98%), high missing genotype call rate (≥4%), high heterozygosity (>30%), gender bias, no satisfying Hardy–Weinberg equilibrium (HWE; *p* < 1 × 10^−5^), and a minor allele frequency (MAF) of <1% [22].

### 2.6. Screening of Genetic Variants and Generation of the Haplotype for 3GO Risk

Genetic variants were selected from genome-wide association study (GWAS) results for the 3GO risk, where GWAS was conducted using EditPlus software after adjusting for age, gender, residence area, and BMI. Genetic variants affecting 3GO risk were selected using *p* < 5 × 10^−6^ since genetic effects were determined by haplotype. Subsequently, SNP-related genes were identified by searching the SCAN annotation database site (http://scandb.org/newinterface/ (accessed on 22 July 2020).). Linkage disequilibria (LD) were determined to check for correlations between SNPs in the selected locus of the chromosome using Locus Zoom (r^2^ value < 0.3).

The haplotype was generated from the selected 5 SNPs in the 11q23 locus, and polygenetic scores (PRS) of the haplotype were estimated by adding the number of risk alleles in each genetic variant. PRS of the haplotype was divided into three groups: 0–3 (major alleles), 4–6 (heterozygous alleles), and ≥7 (minor alleles). Haplotype alleles were used to estimate genetic and genetic × lifetime impacts on 3GO.

### 2.7. Interactions between PRS and Lifestyles on 3GO Risk

The impacts of interactions between genetic variants and lifestyles on 3GO risk were analyzed. Daily energy, protein, fat, carbohydrate, Ca, coffee and alcohol intake, smoking status, and physical activity were included in the interaction analysis as lifestyle variables. Dietary intakes, smoking statuses, and physical activities were dichotomized as ‘high’ or ‘low’ about median values. Residence area, age, gender, BMI, smoking, drinking status, coffee, energy, fat, carbohydrate, cholesterol intakes, physical activity, and plasma total cholesterol concentration were included as covariates in the interaction regression model.

### 2.8. Statistical Analysis

Statistical analysis was performed using SPSS Ver. 24.0 (IBM SPSS Statistics). Descriptive statistics of categorical variables, such as gender and dietary frequency distribution, were obtained using the chi-square test. Descriptive statistics of continuous variables are presented as means and standard deviations (SDs). Covariates for adjustment were as follows: age, gender, area of residence, BMI, daily energy intake, cholesterol intake, fat intake percentages, physical activity, smoking status, and alcohol consumption. Five SNPs in the identified chromosome 11 (the 11q23 locus) were used for haplotype analysis. The major, heterozygous, and minor alleles of the haplotype were scored as 0, 1, and 2, respectively. The Chi-square test was used to analyze the frequency distributions of categorical variables. One-way analysis of variance (ANOVA) was used to determine the significances of differences between the control, 1–2GO, and 3GO groups after covariate adjustment for age, gender, area of residence, and BMI. One-way ANOVA was used to determine the differences between haplotype groups after adjusting for age, gender, residence area, and BMI. Duncan’s test was used to determine multiple comparisons among the groups if ANOVA was significantly different among the groups of each parameter. Multivariate logistic regression analysis controlled for covariates was used to determine the adjusted odds ratio (OR) and 95% confidence interval (CI) of the haplotype for the risk of 3GO and its components. Multivariate interaction regression models were used to evaluate the impacts of interactions between haplotype alleles and lifestyles on 3GO risk, after adjusting for covariates of age, gender, residence area, and BMI (model 1), and those for model 1, physical activity, and energy, alcohol, and coffee intake (model 2). When a significant interaction was found, the lifestyle variable concerned was dichotomized into low and high groups, and in each group, adjusted OR and CI between the haplotype and 3GO risk were estimated. Statistical significance was accepted for *p*-values < 0.05.

## 3. Results

### 3.1. General Characteristics of the Participants

**Figure 1:**
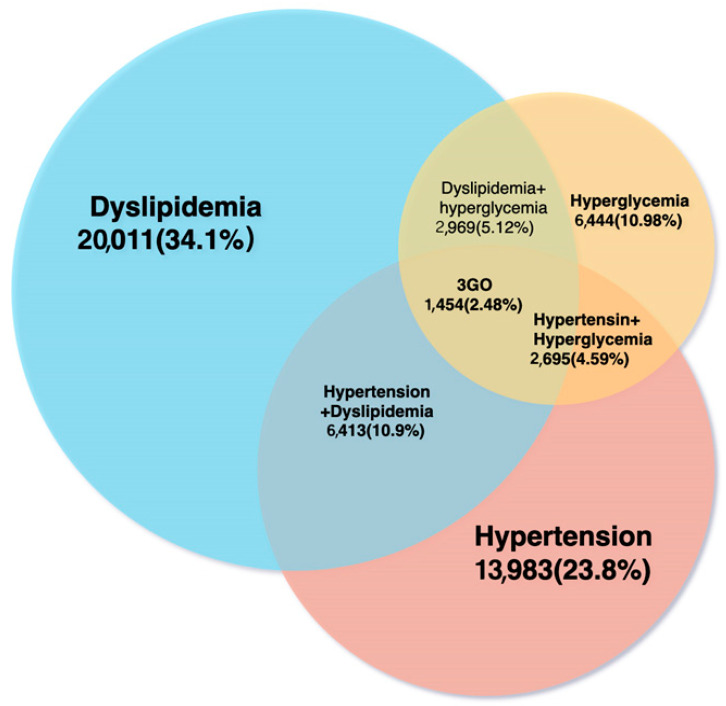
Numbers and percentages of participants with dyslipidemia, hypertension, or hyperglycemia.

Of the 58,701 individuals that participated in KoGES, 1454 (2.48%) had 3GO. In the 2GO group, 2695 (4.59%) had hypertension and hyperglycemia, 6413 (10.9%) had hypertension and dyslipidemia, and 2969 (0.51%) had dyslipidemia and hyperglycemia (Figure 1). A total of 16,730 among 58,701 participants had all or either hypertension, hyperglycemia, or dyslipidemia. The average age of patients in the 3GO group was about seven years greater than that in the control group. Interestingly, most of the variables related to MetS were significantly different in the control, 1–2GO, and 3GO groups. BMI, waist circumferences, hip circumferences, and body fat percentages were reduced in 3GO, 1–2GO, and control groups. Mean waist circumference and body fat percentage were much higher in the 3GO group, by 9.1 cm and 6.2 % respectively, than in the control group (Table 1). Fasting serum glucose concentrations showed that only participants in the 3GO group had a level > 126 mg/dL. Serum lipid profiles showed that participants in the 3GO group had higher serum triglyceride concentrations and lower serum HDL cholesterol concentrations. The average lipid profiles in the 3GO group were lower than the diagnostic criterion since the participants having lipid-lowering medications were included as the 3GO case. Serum triglyceride concentrations showed the most significant differences, and the gap between the 3GO and control groups was 79.7 mg/dL. The mean serum triglyceride concentrations were 1.86 times higher in the 3GO group. SBP and DBP were also much higher in the 3GO group, by 15 and 7.2 mmHg respectively, than the control group (Table 1).

Total physical activity and coffee intake were not significantly different in the three groups, whereas alcohol drinking and smoking were. Interestingly, energy intake, which was calculated as a percentage of estimated energy requirement, was lower in the 3GO group than the other two groups (Table 1). In terms of nutritional intake, participants in the 3GO group had a higher mean carbohydrate intake than those in the other groups, while protein and fat intake in the 3GO group were lower than in the control group. Ca and undigested carbohydrate (dietary fiber) intakes were also lower in the 3GO group than in the other groups (Table 1). These results suggest that the incidences of hypertension, hyperglycemia, and dyslipidemia are positively associated with improper nutrient intake, regardless of energy intake, alcohol intake, and smoking status, and negatively related to exercise.

### 3.2. Selection of Genetic Variants

**Figure 2:**
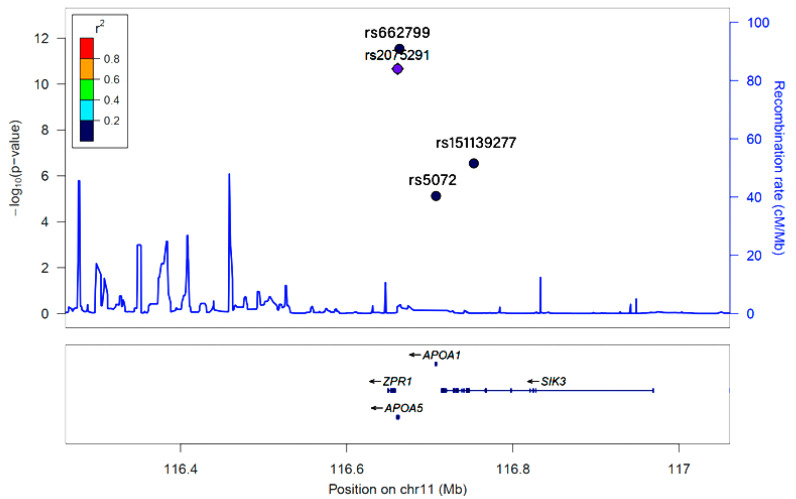
Linkage disequilibrium (LD) of *KCNQ1*_rs2237892, *ZPR1*_rs2075291, *APOA5*_rs662799, *APOA1*_rs5072, and *SIK3*_rs151139277, as determined by Locus Zoom.

After adjusting for age, gender, area of residence, BMI, smoking status, coffee intake, drinking status, total activity, and total cholesterol, 5 SNPs selected from the 11q23 locus were found to influence 3GO risk (Table 2), and all 5 satisfied the Hardy–Weinberg equation (*p >* 0.05) and MAF (>0.1). Four of the 5 SNPs, except rs2237892, were positively associated (OR = 1.202~1.875; *p* = 7.49 × 10^−6^~2.17 × 10^−11^) with 3 GO risk (Table 2). The genes of these genetic variants were related to lipoprotein metabolism, *APOA5* (*apolipoprotein A5*) rs662799, *APOA1* (*apolipoprotein A1*)_rs5072, *ZPR1* (*zinc finger protein*)_rs2075291, and *SIK3* (*SIK family kinase 3)*_rs151139277. No LD was observed among the SNPs (Figure 2). *KCNQ1* (*potassium voltage-gated channel subfamily Q member 1*)_rs2237892 exhibited a negative relationship with 3GO risk (Table 2).

*KCNQ1*_rs2237892, *ZPR1*_rs2075291, *APOA5_rs662799, APOA1_rs5072,* and *SIK3_rs151139277* were found to be associated with 3GO risk by a genome-wide association study of a hospital-based urban cohort of 58,701 individuals. Based on the relationship between 4 SNPs and rs2237892, r^2^ values were represented as color and marker shapes. As shown in the graph, r^2^ colors represented strongest (red; r^2^ > 0.8), strong (orange; r^2^ = 0.8–0.6), moderate (green; r^2^ = 0.4–0.6), weak (sky blue and diamond shape; r^2^ = 0.2–0.4), and weakest (dark blue and round shape; r^2^ < 0.2) between the designated SNP and rs2237892. Five SNPs had a week or weakest relation with each other and had no LD relation.

### 3.3. Adjusted Means of MetS and Its Components According to 3GO and Haplotype Groups

The haplotype was generated by *KCNQ1*_rs2237892, *ZPR1*_rs2075291, *APOA5_rs662799, APOA1_rs5072*, and *SIK3_rs151139277* in 11q23. It showed that 14,317, 13,118, and 1005 in the control participants belonged in the major, heterozygous, or minor alleles of the haplotype (Table 3). On the other hand, in 3GO patients, 210 had the major haplotype, 1131 had a heterozygous haplotype, and 113 had the minor haplotype. Regarding genetic impacts, fasting serum glucose, total cholesterol, triglyceride concentrations, and DBP were significantly higher in the 3GO group than in controls (Table 3). Participants with the haplotype’s minor allele had higher glucose, HbA1c, total cholesterol, and triglyceride concentrations in the blood than those with the major allele (Table 3). Interestingly, BMI was lower in the minor haplotype group than the major haplotype group, although BMI was higher in the 3GO group than in the control group. These results confirmed that the haplotype generated by the *KCNQ1*, *ZPR1*, *APOA5*, *APOA1*, and *SIK3* genes significantly affected 3GO risk and that fasting serum glucose, blood pressure, and serum lipid profiles are significantly associated with 3GO risk.

### 3.4. 3GO-Related Parameters and Their Influences on 3GO Risk According to Haplotype

After adjustment for age, gender, residence area, BMI, physical activity, and energy, alcohol, and coffee intakes, considered as model 2, the minor alleles of haplotype containing *KCNQ1*, *ZPR1*, *APOA5*, *APOA1*, and *SIK3* genetic variants were significantly associated with increased 3GO risk (OR = 3.230, 95% CI = 2.062~5.061, *p* < 0.001). We also analyzed the associations between the haplotype containing *KCNQ1*_rs2237892, *ZPR1*_rs2075291, *APOA5_rs662799, APOA1_rs5072,* and *SIK3_rs151139277* and anthropometric and biochemical parameters. In model 2, the haplotype’s minor alleles were positively associated with SBP, HbA1c, serum glucose, total cholesterol HDL, and triglyceride concentration by 2.135-, 2.688-, 2.138-, 3.095-, 3.196-, and 3.568-fold respectively, as compared with the major alleles of haplotype (*p* < 0.001) (Table 4). However, waist circumferences, DBP, and SBP were not significantly associated with haplotype in models 1 or 2 (Table 4). Although obesity was a significant risk factor for MetS, and BMI was higher in the 3GO group than the control group, BMI was negatively associated with the minor alleles of haplotype by 0.757-fold compared to the haplotype’s major alleles (Table 4).

### 3.5. Haplotype–Environmental Interactions

Table 5 shows the impact of interactions between 11q23 loci and diet or lifestyle on the risk of 3GO. Interactions were observed between haplotype and nutrient intakes, including intakes of protein (*p* = 0.033), carbohydrates (*p* = 0.012), fat (*p* = 0.008), and undigested carbohydrates (dietary fiber; *p* = 0.015). As regards lifestyle factors, haplotype interacted with smoking status to influence 3GO risk (*p* = 0.007). However, alcohol intake, coffee intake, and physical activity did not interact with haplotype to modify the risk of 3GO.

**Figure 3:**
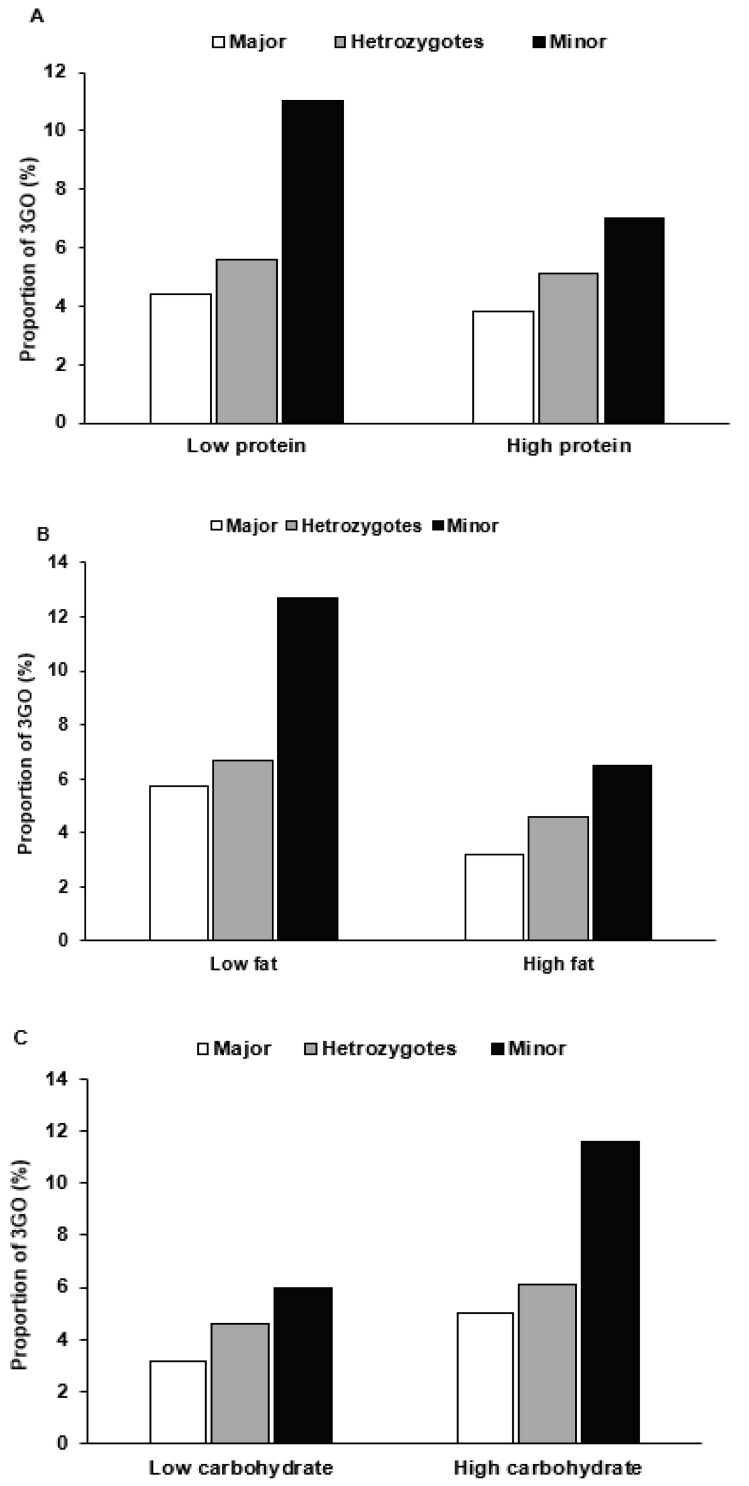

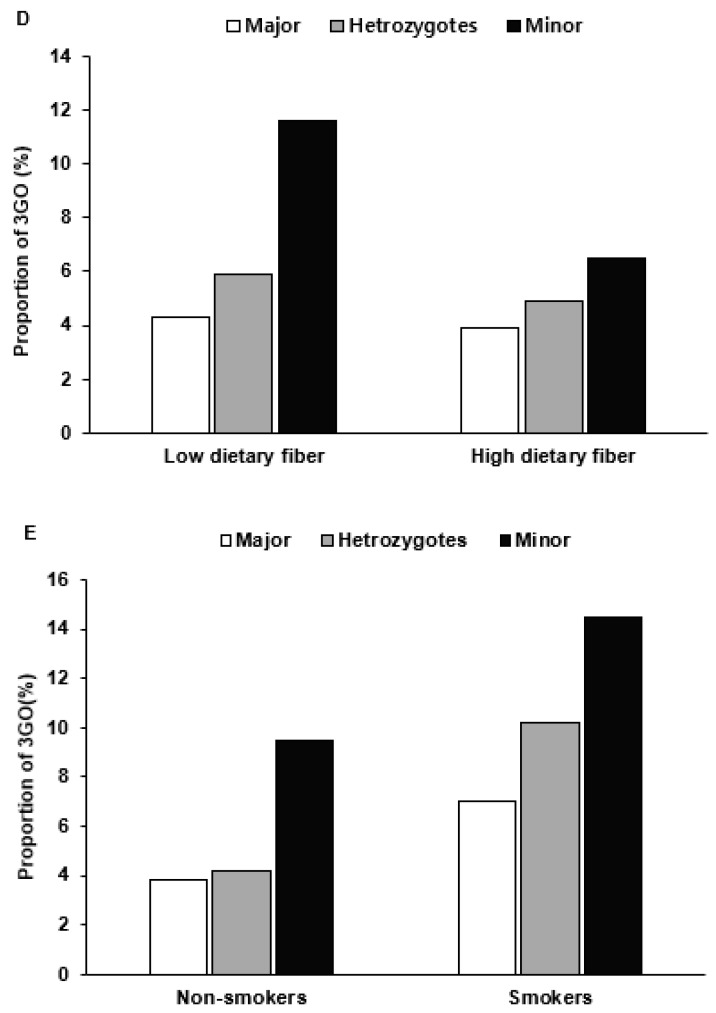
The proportion of participants with the major, heterozygous, and minor alleles of the haplotype in *11q23* according to high- and low-nutrient intake and smoking status. The proportions of 3GO were provided in a low and high intake of protein ((**A**) Cutoff: 13% energy), fat ((**B**) Cutoff: 15% energy), carbohydrate ((**C**) Cutoff: 70% energy), and dietary fiber (undigested carbohydrate; (**D**) Cutoff: 5 g/1000 kcal) intake and smoking status (**E**).

After dichotomizing subjects into low- and high-intake groups, haplotype was found to interact with nutrient intake and 3GO risk. For those with the minor haplotype allele, 3GO risk was increased by 3.3- and 3.2-fold in low-protein and low-fat intakes respectively (Table 5), and for low-protein and low-fat intake, the risk of 3GO was much higher for the minor alleles of haplotype than the major alleles (Figure 3A,B). Unlike that observed for protein or fat intake, the prevalence of 3GO had a higher association with the minor allele of haplotype in high-carbohydrate intake than for low-carbohydrate intake (Table 5), and 3GO frequencies were much higher for the minor allele of haplotype in a high-carbohydrate intake (Figure 3C). In contrast to carbohydrate intake, high dietary fiber (undigested carbohydrates) intake was highly associated with minor allele haplotype compared to low dietary fiber (Table 5). In low dietary fiber intake, 3GO frequencies were much higher in the minor allele than the major allele (Figure 3D). In the smokers and ex-smokers, 3GO risk was associated with the minor allele of haplotype more than in the non-smokers (Table 5), and smokers with the minor alleles had much higher 3GO frequencies (Figure 3E).

## 4. Discussion

Although BMI is low, Asians are more inclined to 3GO than whites [24,25]. This increment may be due to genetic differences and interaction with environmental factors. In the present study, we aimed to explore common genetic variants to influence 3GO risk and find the genetic variants–lifestyle interaction to influence 3GO risk in a city hospital-based cohort in Korean adults ≥ 40 years. To the best of our knowledge, this is the first study to explore the common genetic variants in 11q23 locus for 3GO risk and to show that complex genetic variants and environmental interactions, especially nutrient intakes, contribute to 3GO risk. Traditionally, the Asian diet is based on less refined rice, mostly with vegetables, but Asians consume refined rice with fewer vegetables in the last decades. These dietary pattern changes decrease undigestible carbohydrate (dietary fiber) intake and increase digestible carbohydrate intake. These changes may play a critical role in elevating 3GO risk through SNP–nutrient interactions [26].

Furthermore, Asians and Caucasians also consume different proportions of carbohydrates, fats, and proteins [27]. A very high carbohydrate intake with low dietary fiber and vitamin C is a culprit for metabolic syndrome in Asians [21,22]. The present study shows that 51.6% of 58,701 participants had either hypertension, hyperglycemia, or dyslipidemia in KoGES, and that 2.5% of them satisfied the criteria of 3GO. Participants with 3GO had lower energy intakes, higher proportions of carbohydrates, and lower proportions of fat, protein, and undigested carbohydrates in the diet than those without 3GO. These results indicated that adults need to consume higher proportions of protein, undigested carbohydrates, and adequate amounts of fat to reduce the incidence of 3GO.

*KCNQ1* is a voltage and lipid-gated potassium channel expressed in various tissues, including the myocardium, inner ear, kidney, lung, stomach, and intestines. *KCNQ1* affects insulin secretion, and its mutation is related to the prevalence of T2DM [28,29]. Its polymorphisms, located in intron 15, include rs2237892, rs2237895, and rs2237897, and are significantly related to impaired insulin secretion [30]. The rs2237892 SNP locus has also been related to T2DM susceptibility in East Asian and European populations [26,31]. The present study also showed that KCNQ1 rs2237892 was one of the common genetic variants to influence 3GO risk. *ZPR1* is a cell proliferation and signal transduction regulator protein with various physiological functions [27], and rs964184 of *ZPR1* has been associated with T2DM hypertriglyceridemia in Asians [28]. The 11q23 locus haplotype, which includes *ZPR1* rs2075291, influences MetS risk with serum triglyceride and HDL levels [29], as shown in the present study. Thus, *ZPR1* rs2075291 is associated with hypo-LDL cholesterolemia, hypertriglyceridemia, and hyperglycemia.

The 11q23 locus also encodes *APOA5*, which has been implicated in regulating plasma triglyceride levels and the prevalence of MetS either independently or via complex gene–gene and/or gene–environment interactions. Consistent with the result of the present study, rs662799 of *APOA5* has been reported to increase the risk of MetS in Caucasians [32], Japanese [33], Koreans [34], and Chinese [35,36]. *APOA1* is a primary lipoprotein associated with serum HDL concentrations in T2DM [37], and rs5072 of *APOA1* is related to dyslipidemia in a gender-specific manner, as shown in the present study [38]. *SIK3* plays crucial mediating roles in the three essential cancer signalings: cell proliferation, inflammation, and metastasis [39], and has been related to anti-inflammatory activity [40] and chronic inflammation. Since chronic inflammation is a well-known precursor of MetS development and progression [41], *SILK3* can be associated with MetS risk. The present study shows that *SIK3* rs151139277 is related to 3GO, which suggests that inflammation may be associated with MetS. Summarizing these results, it appears that the 11q23 locus haplotype may be associated with hypertriglyceridemia, hypo-HDL-cholesterolemia, hyperglycemia, and inflammation, and contribute to blood flow disturbances, leading to hypertension.

In this present study, a high carbohydrate intake but low protein, fat, and undigested carbohydrate intakes were associated with significant risks of 3GO, especially in participants with the minor alleles of haplotype. Protein, carbohydrate, fat, and undigested carbohydrate intakes and smoking status interacted significantly with the 11q23 locus haplotype to modulate 3GO risk. Some Asians, including Koreans, consume a white rice-based diet high in carbohydrates but low in protein and fat, and previous studies have shown that Asian adults on a rice-based diet have a high incidence of MetS [22,42,43]. Due to the progression of the grain milling process, undigestible carbohydrate consumption has been reduced in Asians having rice as staple foods. Therefore, Asian adults, especially those with the minor allele of the 11q23 locus haplotype, should be recommended a high intake of dietary fiber, good-quality protein, and omega-3 fatty acid-rich foods, such as fruits, vegetables, and fish.

3GO risk is known to be reduced by an undigested carbohydrates-containing diet [44], as was observed in the present study. Dietary fiber can slow down digestion rates and promote cholesterol and glucose excretions into feces, thus reducing serum glucose and cholesterol levels. Undigested carbohydrate intake can also protect against the development of 3GO and regulate gut microbiota’s metabolic functions to prevent or treat metabolic diseases [45]. In a meta-analysis, individuals that switched from a low-undigested carbohydrate diet (<15 g/day) to a high-undigested carbohydrate diet (25–29 g) were found to have a 1.3% lower mortality rate and a 0.6% lower incidence of heart disease, and to be at lower risk of developing T2DM, hypertension, dyslipidemia, and bowel cancer and of becoming obese [46,47]. Furthermore, undigested carbohydrate intake interacted with haplotype, and haplotype was more associated with 3GO risk in the low-undigested carbohydrate intake group than in the high-undigested carbohydrate intake group.

Tobacco seriously harms cardiovascular health, and thus, helping cardiovascular patients quit smoking and avoid secondhand smoke exposure can improve prognosis, and interestingly, the effect of smoking cessation can be equal or better than single drug treatments for metabolic diseases [48]. Furthermore, smoking elevates the risks of metabolic diseases [49]. We found that the haplotype in the 11q23 locus interacted with smoking to increase 3GO risk, and in smokers, the haplotype had a much stronger association with 3GO risk.

The present study was undertaken to explore common genetic variants associated with 3GO risk and the impacts of interactions between their haplotype at the 11q23 locus and intakes of carbohydrates, protein, fats, and undigested carbohydrate, and smoking status for the risk of 3GO and its components. This study shows that the increased prevalence of 3GO in Asian countries might be linked to genetic factors and their interactions with nutrient intake and smoking status. However, this study has several limitations. (1) The study was conducted using a case-control design, and thus, its results cannot establish causal relationships. (2) The study was conducted on an urban hospital-based cohort recruited from several cities without stratification, which means our results may not represent the Korean population, despite the large number of participants recruited (*n* = 58,701). (3) Nutrient intakes were calculated using self-estimates of food intakes determined by the SQFFQ, and thus, some daily nutrient intakes may have been inaccurate. However, the SQFFQ included 103 foods that Koreans consume frequently and demonstrated good validity and reproducibility compared with 12-day food records [50].

## 5. Conclusions

3GO was found to be associated with high BMIs, waist circumference, elevated fasting serum glucose, total cholesterol, triglyceride levels, systolic and diastolic pressures, and low serum HDL levels in our adult Asian cohort. We also found the haplotype generated from 5 SNPs (*KCNQ1*_rs2237892, *ZPR1*_rs2075291, *APOA5*_rs662799, *APOA1*_rs5072, and *SIK3*_rs151139277) in the 11q23 locus had a 3GO risk. Interactions of haplotype with carbohydrate, fat, protein, and undigested carbohydrate intakes and smoking status influenced 3GO risk. Low intakes of fat, protein, and undigested carbohydrate were positively associated with 3GO risk in individuals with the haplotype’s minor allele. Higher carbohydrate intake and smoking by individuals with the haplotype’s minor allele were also associated with an elevated risk of 3GO. We conclude that individuals having the minor allele of the 11q23 locus haplotype need to prohibit smoking and replace digestible carbohydrates intake into the consumption of undigested carbohydrates (mainly dietary fiber), high-quality protein, healthy fat, and calcium. The results of this study could be applied to prevent 3GO in those at genetically elevated 3GO risk by modifying lifestyles.

## Figures and Tables

**Table 1:** General characteristics of the study population according to 3GO.

	Control	1–2Go	Case (3GO)	*p-*Value ^1^
	(*n* = 28,440)	(*n* = 28,807)	(*n* = 1454)	
Gender (Number, male %)	7712 (27.1)	11,413 (40.7)	722 (49.7)	<0.0001
BMI ^2^ (kg/m^2^)	23.1 ± 2.7 ^c^	24.5 ± 2.9 ^b^	25.8 ± 3.1 ^a^	<0.0001
Waist circumference (cm)	78.2 ± 8.1 ^c^	82.9 ± 8.3 ^b^	87.3 ± 8.4 ^a^	<0.0001
Hip circumference (cm)	93.2 ± 5.6 ^c^	94.8 ± 5.8 ^b^	96.2 ± 6.5 ^a^	<0.0001
Body fat (%)	16.7 ± 7.1 ^c^	17.6 ± 7.0 ^b^	22.9 ± 6.5 ^a^	<0.0001
Serum glucose (mg/dL)	89.7 ± 9.3 ^c^	98.7 ± 22.5 ^b^	132.4 ± 40.3 ^a^	<0.0001
HbA1c (%)	5.53 ± 0.4 ^c^	5.81 ± 0.8 ^b^	7.09 ± 1.3 ^a^	<0.0001
Total cholesterol (mg/dL)	191 ± 26.2 ^b^	204 ± 41.4 ^a^	188 ± 43.9 ^c^	<0.0001
HDL ^3^ (mg/dL)	57.4 ± 11.7 ^a^	50.8 ± 13.5 ^b^	45.7 ± 11.9 ^c^	<0.0001
TG ^4^ (mg/dL)	92.3 ± 37.9 ^c^	144 ± 73.8 ^b^	172 ± 80.7 ^a^	<0.0001
SBP (mmHg)	118 ± 12.6 ^c^	127 ± 15.3 ^b^	133 ± 15.1 ^a^	<0.0001
DBP (mmHg)	73.0 ± 8.5 ^c^	78.3 ± 10.1 ^b^	80.2 ± 10.1 ^a^	<0.0001
Total activity (Number, %)				0.231
None or little (<90 min/w)	12,749 (45.9)	12,739 (46.5)	685 (48.1)	
Moderate (90–150 min/w)	9315 (33.5)	9113 (48.3)	443 (31.1)	
Heavy (>150 min/w)	5724 (20.6)	5550 (20.3)	297 (20.8)	
Alcohol intake (g/day)				<0.0001
Non-drinker (<1)	15,008 (52.9)	14,312 (51.2)	717 (64.1)	
Light drinker (1–15)	782 (2.9)	1302 (4.7)	99 (34.3)	
Moderate drinking (15–30)	8252 (29.4)	8112 (28.9)	422 (29.1)	
Heavy drinker (>30)	4175 (14.8)	4206 (14.7)	211 (14.5)	
Coffee intake (cups/day)				
Non-drinker (0)	4588 (16.2)	4630 (16.5)	262 (18.1)	0.225
Light drinker (<2)	4727 (16.7)	4675 (16.8)	240 (16.6)	
Moderate drinker (2–10)	14,208 (51.1)	13,797 (49.2)	708 (48.9)	
Heavy drinker (>10)	4735 (17.2)	4766 (16.9)	236 (16.4)	
Smoking (Number, %)				<0.0001
Non-smoking	6631 (23.3)	6241 (22.3)	290 (19.9)	
Past-smoking	959 (3.4)	1607 (5.7)	95 (6.5)	
Heavy smoking	676 (2.4)	1121 (4.0)	60 (4.1)	
Energy intake (EER ^5^ percent)	92.2 ± 29.3 ^a^	91.4 ± 28.5 ^b^	89.3 ± 28.5 ^c^	<0.0001
Carbohydrate intake (energy percent)	71.3 ± 7.0 ^b^	72.1 ± 7.0 ^b^	72.8 ± 6.7 ^a^	<0.0001
Protein (energy percent)	13.5 ± 2.5 ^a^	13.4 ± 2.6 ^ab^	13.2 ± 2.5 ^b^	<0.0001
Fat intake (energy percent)	14.3 ± 5.4 ^b^	13.5 ± 5.4 ^a^	12.8 ± 5.2 ^a^	<0.0001
Undigested carbohydrates (g/1000 kcal)	5.74 ± 2.83 ^a^	5.70 ± 2.84 ^a^	5.45 ± 2.65 ^b^	<0.0001
Na (mg/1000 kcal)	2432 ± 1359 ^b^	2446 ± 1404 ^a^	2382 ± 1463 ^c^	0.143
Ca (mg/day)	450 ± 258 ^a^	440 ± 256 ^b^	414 ± 232 ^c^	<0.0001

The values represent adjusted means ± standard deviations or the number of the subjects (percentage of each group). ^1^ The statistical analysis for comparing continuous variables was conducted with one-way analysis of variance (ANOVA) after adjusting for age, sex, residence area, survey year, body mass index (BMI), smoking, physical activity, intake of energy, fat percent, carbohydrate percent, cholesterol, coffee, alcohol, and menopause. The statistical analysis for categorical variables was analyzed with χ^2^ tests. ^a,b,c^ Means without a common letter differ in the same row by Tukey’s test at *p* < 0.05. ^2^ BMI, body mass index; ^3^ HDL, high-density lipoprotein; ^4^ TG, triglyceride; ^5^ EER, estimated energy requirement.

**Table 2:** Characteristics of genetic variants to influence 3GO risk selected by genome-wide association study (GWAS) in a Korean city hospital-based cohort.

Chr ^a^	SNP ^b^	Position	Mi ^c^	Ma ^d^	OR ^e^	*p*_Adjust ^f^	MAF ^g^	*p*_HWE ^h^	Gene	Functional Consequence
11	rs2237892	2839751	T	C	0.8155	1.41 × 10^−6^	0.3755	0.3933	*KCNQ1*	intron variant
11	rs2075291	116661392	A	C	1.585	2.17 × 10^−11^	0.0793	0.3655	*ZPR1*	upstream transcript variant
11	rs662799	116663707	G	A	1.355	2.90 × 10^−12^	0.2988	0.2874	*APOA5*	upstream transcript variant
11	rs5072	116707583	A	G	1.202	7.49 × 10^−6^	0.3593	0.8018	*APOA1*	intron variant
11	rs151139277	116753093	T	C	1.875	2.85 × 10^−7^	0.0192	0.7412	*SIK3*	downstream transcript variant

^a^ Chromosome; ^b^ Single nucleotide polymorphism; ^c^ Minor allele of haplotype; ^d^ Major allele of haplotype; ^e^ Odds ratio of each SNP for 3GO risk; ^f^ *p-*value for ORs after adjusting for age, gender, residence area, and BMI; ^g^ Minor allele frequency; ^h^ Hardy–Weinberg equilibrium.

**Table 3:** Adjusted means and standard deviations according to haplotype allele and control and 3GO groups.

	Control (*n* = 28,440)			3GO (*n* = 1454)		
	Major (*n* = 14,317)	Heterozygote (*n* = 13,118)	Minor (*n* = 1005)	Major (*n* = 613)	Heterozygote (*n* = 744)	Minor (*n* = 97)
BMI ^1^ (kg/m^2^)	23.2 ± 2.71 ^b^	23.1 ± 2.66 ^b^	22.7 ± 2.58 ^b^	25.9 ± 3.12 ^a^	25.7 ± 3.04 ^a^	25.7 ± 3.21 ^a^*^+++^
Waist circumference (cm)	78.4 ± 8.13 ^b^	78.1 ± 8.13 ^b^	76.8 ± 7.89 ^b^	87.4 ± 8.41 ^a^	87.4 ± 8.38 ^a^	85.9 ± 7.77 ^a^**^+++^
Hip circumference (cm)	93.3 ± 5.53 ^b^	93.2 ± 5.61 ^b^	92.6 ± 5.31 ^b^	96.4 ± 6.37 ^a^	96.1 ± 6.52 ^a^	95.9 ± 6.09 ^a+++^
Fasting serum glucose (mg/dL)	89.6 ± 9.29 ^c^	89.8 ± 9.29 ^c^	90.1 ± 9.23 ^c^	132.6 ± 44.7 ^b^	131.5 ± 35.7 ^b^	138.9 ± 46.0 ^a^*^+++^
HbA1c	5.49 ± 0.36 ^b^	5.49 ± 0.35 ^b^	5.49 ± 0.36 ^b^	7.19 ± 1.38 ^a^	7.21 ± 1.28 ^a^	7.24 ± 1.53 ^a^*^+++^
Total cholesterol (mg/dL)	190 ± 26.3 ^b^	191 ± 26.1 ^ab^	191 ± 27.1 ^ab^	189 ± 44.3 ^bc^	186 ± 43.7 ^c^	193 ± 43.8 ^a^**^+++^
HDL ^2^ (mg/dL)	58.1 ± 11.9 ^a^	56.9 ± 11.6 ^a^	54.3 ± 10.8 ^ab^	46.6 ± 12.5 ^c^	45.2 ± 11.5 ^c^	44.3 ± 11.7 ^c^***^+++^
TG ^3^ (mg/dL)	88.5 ± 36.9 ^c^	95.4 ± 38.4 ^c^	104 ± 39.9 ^c^	158 ± 75.8 ^c^	181 ± 81.3 ^b^	194 ± 91.8 ^a^***^+++^
SBP ^4^ (mmHg)	118 ± 12.5 ^b^	118 ± 12.6 ^b^	117 ± 12.7 ^b^	132 ± 14.7 ^a^	133 ± 15.4 ^a^	134 ± 14.5 ^a+++^
DBP ^5^ (mmHg)	73.1 ± 8.48 ^c^	72.9 ± 8.47 ^c^	72.6 ± 8.48 ^c^	80.1 ± 9.69 ^b^	80.4 ± 10.3 ^ab^	81.4 ± 9.68 ^a+++^

^1^ BMI, body mass index; ^2^ HDL, high-density lipoprotein; ^3^ TG, triglyceride; ^4^ SBP, systolic blood pressure; ^5^ DBP, diastolic blood pressure. Haplotype was generated by *KCNQ1*_rs2237892, *ZPR1*_rs2075291, *APOA5*_rs662799, *APOA1*_rs5072, and *SIK3*_rs151139277. Major, heterozygote, and minor alleles of haplotype were divided into 3 categories (0–3, 4–6, and >7) by tertiles as the major, heterozygote, and minor alleles, respectively. Adjusted means were calculated after adjusting for age, gender, residence area, BMI, energy, alcohol, and coffee intake, and physical activity. * Significantly different from major allele group at *p* < 0.05; ** *p* < 0.01; *** *p* < 0.001. ^+^ Significantly different by 3GO at *p* < 0.05; ^+++^*p* < 0.001. ^a,b,c^ Means without a common letter differ in the same row by Tukey’s test at *p* < 0.05.

**Table 4:** Adjusted odds ratios for the risk of 3GO and related parameters according to the allele of the haplotype of 11q23 loci after covariate adjustments.

	Model 1			Model 2	
	Major (*n* = 14,930)	Heterozygote (*n* = 13,862)	Minor (*n* = 1102)	Heterozygote (*n* = 13,862)	Minor (*n* = 1102)
3GO	1	1.435 (1.274–1.616) ***	2.938 (2.291–3.768) ***	1.251 (1.009–1.548) *	3.230 (2.062–5.061) ***
BMI	1	0.959 (0.907–1.014)	0.762 (0.651–0.892) **	0.965 (0.910–1.024)	0.757 (0.541–0.893) ***
Waist circumference	1	1.071 (0.966–1.191)	0.791 (0.578–1.082)	1.019 (0.849–1.223)	0.709 (0.401–1.256)
SBP	1	1.134 (1.014–1.268) *	1.689 (1.307–2.183) ***	1.090 (0.877–1.354)	2.135 (1.328–3.432) **
DBP	1	1.171 (1.022–1.342) *	1.435 (1.029~2.002) *	1.188 (0.949–1.487)	1.039 (0.548–1.971)
BP	1	1.171 (1.022~1.342) *	1.435 (1.029~2.002) *	1.121 (0.943~1.333)	1.487 (0.966~2.289)
Serum glucose	1	1.123 (1.052~1.199)	1.478 (1.255~1.740) ***	1.141 (0.888~1.466)	2.138 (1.222~3.742) **
HbA1c	1	1.374 (1.214–1.556) *	2.670 (2.056–3.467) ***	1.365 (1.190–1.544) *	2.688 (2.048–3.529) ***
Serum total cholesterol	1	1.115 (1.025–1.212)	1.448 (1.183–1.771) **	0.940 (0.593–1.490)	3.095 (1.374–6.972) **
Serum HDL	1	1.320 (1.239–1.406) ***	2.339 (2.020–2.708) ***	1.318 (0.975–1.781) *	3.196 (1.761–5.801) ***
Serum TG	1	1.530 (1.421–1.648)	2.744 (2.330–3.230) ***	2.212 (1.535–3.187) ***	3.658 (1.755–7.624) ***

Values represent odds ratio and 95% confidence intervals after adjusting for covariates age, gender, residence area, and BMI (model 1) and covariates for model 1, plus intake of energy, alcohol, and coffee, physical activity (model 2). Haplotype generated with 5 single nucleotide polymorphisms (SNPs) was divided into 3 categories (0–3, 4–6, and 7–10) by tertiles as the major, heterozygote, and minor alleles, respectively. Haplotype’s major allele was the reference for both model 1 and model 2. * Significantly different from the major allele in logistic regression analysis at *p* < 0.05, ** *p* < 0.01, *** *p* < 0.001.

**Table 5:** Adjusted 3GO for the risk of 3GO and related parameters according to the haplotype of *KCNQ1_rs2237892*, *ZPR1_rs2075291*, *APOA5_rs662799*, *APOA1_rs5072*, and *SIK3_rs151139277*, and interaction with lifestyles.

	Major (*n* = 14,930)	Heterozygote (*n* = 13,862)	Minor (*n* = 1102)	Haplotype–Nutrient Interaction *p-*Value
Low energy High energy	1	1.383 (1.219~1.569) *** 1.926 (1.338~2.773) ***	2.853 (2.198~3.701) *** 3.877 (1.675~8.978) **	0.533
Low protein High protein	1	1.383 (1.171~1.633) *** 1.486 (1.254~1.762) ***	3.261 (2.331~4.563) *** 2.559 (1.762~3.716) ***	0.033 *
Low carbohydrate High carbohydrate	1	1.622 (1.344~1.957) *** 1.316 (1.128~1.535) ***	2.640 (1.741~4.004) *** 3.111 (2.277~4.248) ***	0.012 *
Low fat High fat	1	1.231 (1.039~1.459) ** 1.648 (1.395~1.947) ***	3.153 (2.234~4.450) *** 2.721 (1.894~3.909) ***	0.008 *
Low dietary fiber High dietary fiber	1	1.462 (1.234~1.734) *** 1.409 (1.193~1.664) ***	3.673 (2.629~5.122) *** 2.271 (1.554~3.319) ***	0.015 *
Low alcohol High alcohol	1	1.296 (1.106~1.520) ** 1.628 (1.359~1.951) ***	2.763 (2.001~3.817) *** 3.126 (2.110~4.630) ***	0.786
Low exercise High exercise	1	1.753 (1.391~2.211) *** 1.354 (1.044~1.756) *	2.413 (1.663~3.501) *** 3.129 (1.812~5.403) ***	0.959
Low coffee High coffee	1	1.479 (1.206~1.813) *** 1.413 (1.235~1.658) ***	2.751 (1.776~4.258) *** 3.062 (2.262~4.145) ***	0.924
Non-smoker Smoker + ex-smoker	1	1.119 (0.861~1.455) 1.632 (0.887~3.002)	3.365 (2.012~5.630) *** 5.283 (1.408~19.82) **	0.007 *

Values represent the odds ratio and 95% confidence intervals after adjusting for age, gender, residence area, BMI, intake of energy, alcohol, and coffee, and physical activity without the corresponding variables. Haplotype was generated with *KCNQ1* (*potassium voltage-gated channel subfamily Q member 1*)_rs2237892, *ZPR1* (*ZPR1 zinc finger)*_rs2075291, *APOA5* (*apolipoprotein A5)*_rs662799, *APOA1* (*apolipoprotein A1*)_rs5072, and *SIK3* (*SIK family kinase 3*)_rs151139277. Haplotype was divided into 3 categories by tertiles as the major, heterozygote, and minor alleles. Reference was the major allele of the haplotype. Criteria of low and high definition of each parameter in interaction analysis: daily consumption less than estimated energy intake, less than 13% protein, 70% carbohydrate, and 15% fat of daily energy consumption, alcohol drinking > 20 g, coffee intake > 1 cup/day, and 90 min/day moderate physical activity. * Significantly different from major allele in logistic regression analysis at *p* < 0.05, ** *p* < 0.01, *** *p* < 0.001.

## Data Availability

The data presented in this study are available on request from the corresponding author.
